# Commentary: Big data bring big controversies: HDL cholesterol and mortality

**DOI:** 10.1093/ije/dyab016

**Published:** 2021-02-19

**Authors:** Mika Ala-Korpela, Sanna Kuusisto, Michael V Holmes

**Affiliations:** 1 Computational Medicine, Faculty of Medicine, University of Oulu and Biocenter Oulu, Oulu, Finland; 2 Center for Life Course Health Research, University of Oulu, Oulu, Finland; 3 NMR Metabolomics Laboratory, School of Pharmacy, University of Eastern Finland, Kuopio, Finland; 4 Medical Research Council Population Health Research Unit, University of Oxford, Oxford, UK; 5 Clinical Trial Service Unit & Epidemiological Studies Unit, Nuffield Department of Population Health, University of Oxford, Oxford, UK; 6 National Institute for Health Research Oxford Biomedical Research Centre, Oxford University Hospital, Oxford, UK

No matter how large a study, how richly it is phenotyped or how intriguing the findings that emerge, it behoves us to be sceptical in interpreting evidence arising from observational epidemiological studies. Various intractable sources of error, including reverse causality and residual confounding, hinder interpretations on potential causal relationships between exposures and outcomes in observational study settings.^1^

High-density lipoprotein cholesterol (HDL-C) serves as an excellent cautionary example on over-reliance on observational epidemiology when studying and interpreting the potential clinical role of a biomarker. For decades, in a plethora of observational epidemiological studies, HDL-C has shown consistent inverse associations with cardiovascular disease. However, both Mendelian randomization analyses and randomized controlled trials have recently shed light on this area of particularly confounded findings. Multiple HDL-C raising clinical trials have either failed or demonstrated benefit unrelated to increases in circulating HDL-C, in accordance with genetic findings.^1–4^

Large-scale observational data have recently provided an opportunity to examine potential non-linearities in the association of HDL-C with all-cause mortality—a U-shaped association has emerged as a unanimous feature from several large-scale studies set in diverse populations, suggesting that both low and high circulating HDL-C concentrations might pose as unfavourable to human longevity. The first two such large-scale studies were published in 2016.^5^^,^^6^ In 1 764 986 men, with sensitivity analyses in 82 422 women providing consistent results, Bowe *et al*.^5^ showed a U-shaped association between HDL-C and all-cause mortality (average age 64 years and follow-up 9.1 years). Analyses of the same outcome in the CANHEART research database with 631 762 participants (55% women) recapitulated the same U-shaped association of HDL-C (age 57 years and follow-up 4.9 years).^6^ Analysis using cause-specific mortality indicated that lower HDL-C was associated with higher risk of cardiovascular, cancer and other mortality, and higher HDL-C with higher risk of non-cardiovascular mortality. Based on 52 268 men and 64 240 women from the Copenhagen General Population Study and the Copenhagen City Heart Study combined (age ∼57 years, follow-up 6.0 years), Madsen and co-workers^7^ found that the association between HDL-C and all-cause mortality was U-shaped, the association with very high HDL cholesterol being most pronounced in men and for cardiovascular mortality. Similarly, in 365 457 South Koreans (53% women, age 56 years, follow-up 3.5 years) a U-shaped relationship with HDL-C and all-cause mortality was also detected.^8^ Studies with less than 100 000 participants have also reported compatible findings regarding all-cause mortality.^9^^,^^10^

An extensive study by Yi *et al*.^11^ in this issue of the *International Journal of Epidemiology* examined the relationship of HDL-C and all-cause mortality in 15 860 253 Korean adults (48% women, age 47 years and follow-up 8.6 years), and indicated a clear U-shaped association for both men and women. As an important corollary of the vast number of participants, the authors were able to study several age groups separately. The U-shaped relationship between HDL-C and all-cause mortality was present for all age groups between 18 and 64 years and there was a tendency for the associations to be stronger for men than women. However, particularly at high HDL-C concentrations, the associations attenuated for older individuals between 65 and 99 years of age.

Unfortunately, cause-specific mortality data were not available for these participants. This hampers attempts to further explore the findings in order to understand what drives the elevated comparative all-cause mortality at low and high (versus ‘average’) circulating HDL-C concentrations. It is quite possible that the U-shape has arisen due to discrepant associations of HDL-C with different causes of death—the critical question is whether such associations represent cause and effect relationships or whether they have arisen due to pervasive confounding.^6–9^ The combined effects of multiple confounders, each of which displays a different effect on cause-specific mortality, potentially drives the U-shape relationship of HDL-C with overall mortality. Non-cancer plus non-cardiovascular deaths accounted for 50% of the mortality in Korea during 2003–18, highlighting the potential for disease-specific associations driving the U-shape. The lack of resolution on cause-specific mortality also limits the temporal and translational value of the findings, since the U-shape may well be sensitive to changes in the contemporaneous causes of death. Therefore, as so often is the case with observational epidemiology, these intriguing results, even though derived from a vast number of individuals in this particular study, pose a double-edged sword with neither an apparent explanation nor an opportunity for intervention.

Yi *et al*.^11^ rightly recognize that the observational nature of their study precludes causal deductions. However, we would like to reflect on the nomenclature, with specific reference to the authors’ use of ‘optimal range’ or ‘optimal levels’ which appears multiple times in the study to describe HDL-C concentrations associated with the lowest mortality in their dataset. This should not be construed as implying causation nor should the findings be interpreted to mean that changing HDL-C to a value within the ‘optimal range’ will lead to a modification in the risk of death.

Low and high HDL-C concentrations associate with a higher risk of cardiovascular as well as non-cardiovascular mortality, making circulating HDL-C more of a marker of general health than a modifiable risk factor for cardiovascular disease.^6^ The well-characterized inverse correlation between HDL-C and circulating triglycerides at the population level also adds to the complexity of interpreting HDL-C associations and, in this context, triglycerides are a likely confounder for the observed higher (cardiovascular) mortality at low HDL-C.^12^^,^^13^ In addition, a new reverse remnant-cholesterol transport (RRT) hypothesis has been recently postulated to explain the U-shaped association of HDL-C and cardiovascular mortality.^14^ The RRT hypothesis postulates that an essential HDL function would be to take up and carry away circulating remnant particle cholesterol and thereby reduce cholesterol influx into the arterial wall. The functional property of HDL in connection to RRT is hypothesized to be impaired for both low and high HDL-C.^14^ Whereas this hypothesis cannot account for the U-shaped association of HDL-C as related to non-cardiovascular mortality, but it is thought-provoking that this metabolic hypothesis is compatible with the recently genetically substantiated key role of apolipoprotein B-containing lipoprotein particles in the development of atherosclerosis.^15^

The results of Yi *et al*.^11^ leave little doubt as to the presence of an observational U-shaped association between circulating HDL-C concentrations and all-cause mortality. This study adds a great deal of observational evidence with by far the largest study population. There remains the possibility that HDL-C may play a role in aetiology of non-cardiovascular diseases, e.g. evidence points to a potential protective role in type 2 diabetes.^16^ Nonetheless, whereas such reliable information on the potential causal role of HDL-C remains relatively scarce, the most likely explanation is pervasive confounding, including socioeconomic circumstances, lifestyle, obesity and diet as well as various comorbidities like diabetes and kidney disease.^6^^,^^12^^,^^17^

## Funding

M.A.K. is supported by a research grant from the Sigrid Juselius Foundation, Finland. M.V.H. is supported by a British Heart Foundation Intermediate Clinical Research Fellowship (FS/18/23/33512) and the National Institute for Health Research Oxford Biomedical Research Centre.

## Conflict of Interest

The authors report no conflicts of interest.
